# A Novel *Campylobacter jejuni* Sequence Type from a Culture-Negative Patient in The Gambia

**DOI:** 10.1371/journal.pone.0001773

**Published:** 2008-03-12

**Authors:** Gerard A. J. Morris, Usman N. Ikumapayi, Martin Antonio, Stephen R. C. Howie, Richard A. Adegbola

**Affiliations:** Bacterial Diseases Programme, Medical Research Council Laboratories, Fajara, The Gambia; University of California Merced, United States of America

## Abstract

The introduction of molecular diagnostic methods is crucial for improved understanding of the aetiology and epidemiology of bacterial infections in communities in resource poor settings. A blood sample from a 7 month old patient diagnosed with malaria in 2001 in a Gambian outpatient clinic was reported as culture negative after it was subjected to traditional bacterial culture protocols. We re-addressed the analysis of the blood sample from this case more recently (after 6.5 years in archival storage) in pilot work establishing *16S rRNA* PCR in our molecular laboratory. Initial *16S rRNA* PCR results confirmed the presence of bacterial DNA in the sample. *16S rRNA* sequence analysis identified the organism as *Campylobacter* spp. In light of the molecular evidence we successfully grew the organism using appropriate culture conditions and subsequently biochemically confirmed that the isolate was *Campylobacter jejuni*. PCR and DNA sequencing of a set of seven *C. jejuni* housekeeping genes and *in silico* Multilocus Sequence Typing (MLST) analysis revealed that the isolate exhibits a novel sequence type (ST) of *C*. *jejuni* (ST 2928) and belongs to ST-443 clonal complex. This study demonstrates the potential for molecular tools to enhance the diagnosis of bacterial infections, which remain a major killer globally, not least in children in the developing world. Improvements in diagnostics are needed, and will be important not only for sick individuals but also for populations, where better measures of disease burden will contribute significantly to the improvement of public health policy.

## Introduction

The emergence of opportunistic pathogens in immunocompromised hosts, such as HIV-infected patients, has increased the burden on clinical diagnostic laboratory services in developing countries. Reliable and accurate identification of these pathogens poses a serious challenge, necessitating the widening of the scope of the assays traditionally employed in resource poor settings. Traditional bacterial culturing techniques have many deficiencies, necessitating a search for adjunctive or alternative approaches such as molecular protocols. Errors in identifying poorly described, rarely isolated, or phenotypically aberrant bacterial strains are likely to be common in routine clinical diagnostic laboratories using traditional culturing and biochemical methods exclusively.

Traditional bacterial culturing protocols rely on replicating *in vitro* the physical and biochemical requirements of each bacterial species. The traditional culturing methods are limiting by definition, *e.g.* in a mixed microbial population specific culture media may enrich for a single bacterial species, while a distinct species with distinct physical and metabolic requirements will not be detected despite being present in the sample. The limitations of traditional bacterial culturing technologies have been highlighted by the low culture success rate from samples taken at foci of infection, *e.g.* lung aspirate samples in pneumonia patients [1].

Molecular methods have many advantages over traditional protocols employed for identification and characterization of bacteria in clinical samples, reviewed [2], [3]. Molecular protocols have the potential to rapidly identify the aetiological agents of unexplained, culture-negative illnesses or to resolve or better characterize ‘overlap’ clinical syndromes facilitating more appropriate and more directed therapy. Molecular protocols may play an important role in epidemiological studies where failure of culture-dependent methods to detect low level bacterial carriage compromises the study of changes in microbial ecology in patients following vaccination.

‘Broad-range’ *16S rRNA* PCR and DNA sequencing have emerged as potential adjunctive tools or as alternative culture-independent methods for detecting and identifying pathogenic bacteria in clinical samples. Organism specific MLST analysis allows more definitive identification of bacterial isolates providing useful epidemiological data for identifying the source and course of outbreaks. The extensive and rapidly growing internet based *16S rRNA* and MLST databases facilitate rapid identification of sequences to the species or genus level. The advantages and caveats associated with this broad range PCR approach have been reviewed elsewhere [4]–[5]

‘Overlap’ syndromes, where different diseases are difficult to distinguish clinically, create a challenge for both health workers and health systems, especially in resource-poor countries. This is particularly so in malaria endemic settings where clinical symptoms of malaria are practically indistinguishable from those of pneumonia and can compromise the process of case management strategy [6]. We outline how the application of a range of molecular tools applied to an archival blood sample followed by the utilization of internet bioinformatic resources helped to characterize a novel ST of *C*. *jejuni* in the Gambia. Our results make a strong case for the routine use of molecular techniques, which if applied prudently can play a vital role in clinical diagnosis and monitoring of antibacterial therapy, while retaining cost effectiveness in resource poor developing countries.

## Methods

Informed written consent was obtained as described elsewhere [7]. The study and all protocol revisions were approved by the ethics committees of the Gambia Government/MRC, the London School of Hygiene and Tropical Medicine and by the WHO Secretariat Committee for Research Involving Human Subjects.

Forty seven clinical samples (all lung aspirate and venous blood) from 33 patients were retrieved from our archives; these samples had been stored from this previous study [7]. These samples were selected for a pilot study, undertaken to establish *16S rRNA* PCR in our molecular laboratory. Samples were blinded on assignment of a laboratory number before being processed in batches for DNA extraction and *16S rRNA* PCR. Two of the blood samples (sample numbers 2 and 3) in addition to being positive for *16S rRNA* PCR showed unusual eletrophoretic shifts on agarose gel eletrophoresis, relative to the expected size of the PCR products (see results section below). This aroused our suspicion that the samples might contain unusual bacterial species, and it was on this basis that we pursued these samples for further molecular characterisation. Lack of availability of MLST PCR and sequencing primers, however, restricted our molecular characterisation of the bacterium present to just one of these blood samples, sample number 2, which is highlighted in this case report.

### The patient behind the sample

A whole blood sample had been taken from a 7 month old girl recruited in 2001 as part of a previously described study [7]. The child presented with a several day history of cough, fever and diarrhoea and was not on any medication at the time of assessment. She appeared sick, had a temperature of 39.3°C and had a raised respiratory rate of 46 per minute without signs of severe respiratory distress. A blood film showed a significant *Plasmodium falciparum* parasitaemia (200 parasites per high-powered field). She was admitted to hospital with a diagnosis of malaria, and treated according to local guidelines with chloroquine and pyrimethamine-sulfadoxine. A blood culture taken on admission was reported as negative. She was discharged after 2 days having made a good recovery.

A blood sample from this case was retried from archival storage (−80°C for 6.5 years) and examined by molecular methods for the presence of bacterial DNA as part of a molecular pilot study, as indicated above.

### Extraction of DNA from the sample

100 µl of each of the archival blood samples was added to 180 µl of lysozyme buffer (20 mM Tris-HCl, 2mM EDTA, 1.2 % Triton X-100, 20 mg/ml lysozyme (Sigma)) for 30 min at 37°C. Proteinase K (25 µl) was added and the sample incubated at 70°C for 30 min. The sample was subjected to the standard Qiagen DNA Mini Kit (Qiagen Ltd. UK) extraction protocol with 2×25 µl elutions on the same column using the kit elution buffer. The DNA eluate was stored at −20°C prior to *16S rRNA* PCR.

### *16S rRNA* standard PCR conditions

Each 25 µl *16S rRNA* PCR reaction contained 17.5 µl of molecular biology-grade water (Sigma Aldrich Company Ltd.), 2.5 µl of 10×PCR buffer (Qiagen Ltd.), 1 µl of 12.5 µM each forward and reverse PCR primers, 1 µl of a 10 mM dNTPs (Invitrogen Ltd.), 0.25 µl of *Taq* DNA polymerase (Qiagen Ltd.), and 2 µl of DNA extracted from the clinical sample. Non-template control (NTC) samples contained the same PCR reagents as above but had 2 µl of water substituted for template. The positive control was a local isolate of *Streptococcus pneumoniae*. PCR amplification conditions on an MJ thermocycler were as follows: 94°C for 10 min, followed by 25 cycles of 94°C for 30 s, 60°C for 1 min, and 72°C for 1 min, with a final extension at 72°C for 10 min and storage at 4°C.

### MLST was performed as follows

Seven PCR amplicons were prepared from purified DNA extracted from a glycerol stock of the bacterial isolate by using the PCR primers shown in **Table 1**. Each 25 µl PCR mixture was identical to the *16S rRNA* PCR above, except for the primers and 2 µl of purified *C. jejuni* DNA as template. The amplification conditions were 94°C for 10 min, followed by 35 cycles of 94°C for 30 s, 50°C for 1 min, and 72°C for 1 min, with a final extension at 72°C for 10 min and storage at 4°C. Non-template control (NTC) samples contained the same reagents as above for each of the 7 distinct PCR reactions but had 2 µl of water substituted for template in each case.

**Table 1:** MLST alleles, GenBank accession numbers, PCR and sequencing primers (5′ to 3′) for blood samples No. 2 & 3.

Gene	MLST Allele	Genbank	PCR Forward 5′-3′	PCR Reverse 5′-3′
*aspA*	24	EU056829	AGTACTAATGATGCTTATCC	ATTTCATCAATTTGTTCTTTGC
*glnA*	2	EU056830	TAGGAACTTGGCATCATATTACC	TTGGACGAGCTTCTACTGGC.
*gltA*	2	EU056831	GGGCTTGACTTCTACAGCTACTTG	CCAAATAAAGTTGTCTTGGACGG
*glyA*	15	EU056832	GAGTTAGAGCGTCAATGTGAAGG	AAACCTCTGGCAGTAAGGGC
*pgm*	294	EU056833	GCAAACTCAGGACACCCAGG	AAAGCATTGTTAATGGCTGC
*tkt*	3	EU056834	GCTTAGCAGATATTTTAAGTG	AAGCCTGCTTGTTCTTTGGC
*uncA*	12	EU056835	ATGGACTTAAGAATATTATGGC	ATAAATTCCATCTTCAAATTCC.
*C. jejuni 16S rRNA*	N/A	EU056828	TCCTACGGGAGGCAGCAGT	GGACTACCAGGGTATCTAATCCTGTT
*Tricococcus sp. 16S rRNA*	N/A	EU418263	TCCTACGGGAGGCAGCAGT	GGACTACCAGGGTATCTAATCCTGTT
**Gene**	**MLST Allele**	**Genbank**	**Sequencing Forward 5′-3′**	**Sequencing Reverse 5′-3′**
*aspA*	24	EU056829	CCAACTGCAAGATGCTGTACC	TTCATTTGCGGTAATACCATC.
*glnA*	2	EU056830	CATGCAATCAATGAAGAAAC	TTCCATAAGCTCATATGAAC
*gltA*	2	EU056831	CTTATATTGATGGAGAAAATGG	CCAAAGCGCACCAATACCTG
*glyA*	15	EU056832	AGCCTAATTCAGGTTCTCAA	AGGTGATTATCCGTTCCATCGC
*pgm*	294	EU056833	GGTTTTAGATGTGGCTCATG	TCCAGAATAGCGAAATAAGG
*tkt*	3	EU056834	GCTTAGCAGATATTTTAAGTG	AAGCCTGCTTGTTCTTTGGC
*uncA*	12	EU056835	AAAGTACAGTGGCACAAGTGG	TGCCTCATCTAAATCACTAGC
*C. jejuni 16S rRNA*	N/A	EU056828	TCCTACGGGAGGCAGCAGT	GGACTACCAGGGTATCTAATCCTGTT
*Tricococcus sp. 16S rRNA*	N/A	EU418263	TCCTACGGGAGGCAGCAGT	GGACTACCAGGGTATCTAATCCTGTT

All amplicons from *C.jejuni* isolate unless stated otherwise.

All *16S rRNA* PCR, MLST PCR and DNA sequencing oligonucleotide primers were obtained from Sigma Genosys (**Table 1**).

### Agarose gel electrophoresis

All standard *16S rRNA* PCR and *C. jejuni* specific MLST PCR products were assessed for expected PCR product size on 2 % (w/v) agarose gels with a 100 bp DNA ladder (New England Biolabs). Aliquots of PCR products were electrophoresed at 150V for 45 min; DNA was visualized using ethidium bromide and photographed after UV transillumination with PD-Quest 7.3.1 basic 2D analysis software (Bio-Rad).

### *16S rRNA* and MLST gene sequencing

*16S rRNA* and *C. jejuni* specific MLST PCR products were sent to Macrogen in South Korea (http://www.macrogen.com) for purification and DNA sequencing. For primers used in PCR and sequencing reactions see **Table 1:**

### Bioinformatic applications

All forward and reverse sequences were aligned for each amplicon using the ClustalW program to assess the specificity of the sequencing reactions (http://www.ebi.ac.uk/clustalw/). Reverse sequences were reverse complemented (http://bioinformatics.org/sms/rev_comp.html) prior to alignment using ClustalW**.**

All sequence data was subjected to BLAST analysis (non-redundant nucleotide sequence database search) (http://www.ncbi.nlm.nih.gov/BLAST/) to definitively identify each respective 16S rRNA and MLST gene amplicon.

MLST alleles and sequence types were assigned to the isolate by comparing data to the *Campylobacter* MLST database (http://campylobacter.mlst.net). Data for the newly described alleles and STs were deposited in the *Campylobacter* MLST database http://pubmlst.org/campylobacter/. All resulting gene sequences from this study were deposited in Genbank (**Table 1**).

### Microbiological culture, isolation and biochemical analysis of *C*. *jejuni* from the sample

A 25 µl aliquot of the archival blood sample was inoculated and streaked on *Campylobacter*-selective agar plates Columbia agar (cat # Oxoid CM 0331) prepared aseptically with supplement (Oxoid SR0098E) in 10% sheep blood agar. The cultured plate was incubated in a microbiological culture jar, 10% CO_2_, 5% Oxygen and 85% Nitrogen, achieved using a Campy-gas generating kit (cat # Oxoid BR0060A) and a palladium catalyst. The jar was closed tightly and incubated for 48 hrs at 42°C. Isolates were biochemically characterized using Oxidase cytochrome (N,N,N –Tetramethyl-P-Phenylenediamine dihydrochloride) (Sigma), Catalase (Riedel-de Haen) and Ninhydrin (BactiDrop Ninhydrin, Remel) tests were all carried out for definitive and differential biochemical characterization.

## Results

### *16S rRNA* PCR and sequencing analysis

**Figure 1:**
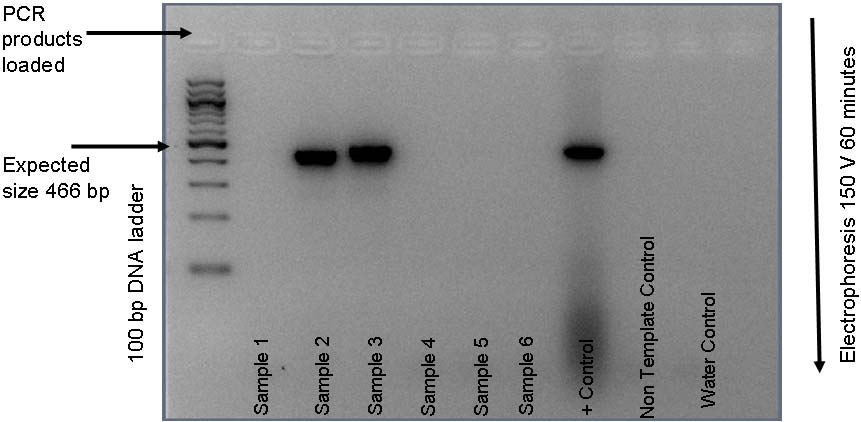
2 % agarose gel, *16S rRNA* PCR of gDNA extracted from archival blood samples 1 to 6. 2 µl of gDNA in 25 µl PCR reaction with 25×PCR cycles. 5 µl loaded on gel. Image is reverse colour under UV transillumination (Bio-Rad). Positive control (+Control) used 2 µl of gDNA from a local *Streptococcus pneumoniae* isolate. Non template control (NTC) consisting of 25 µl PCR mix only, water control 2 µl molecular grade water in place of gDNA.

Of the first batch of six archival clinical sample gDNA extracts, only two were positive for bacterial DNA by *16S rRNA* PCR, sample 2 and 3 (**Figure 1**), these samples represented venous blood samples from 2 distinct patients. Samples 1, 4, 5 and 6 tested negative for bacterial DNA. The gel mobility shift of the 16S rDNA band from the archival blood samples 2 and 3 were visibly distinct in size from the positive control (*Streptococcus pneumoniae*). This observation is based on an expected amplicon size of 466 bp from PCR primers designed to generate products between nucleotide residues 331 and 797 of the *Escherichia coli* 16S rRNA gene [8]. The non-template control samples were negative and the positive control sample was positive as expected. Sequencing of the 16S rRNA PCR products resulted in clear, clean sequence traces for both forward and reverse reactions from sample 2 and 3. There was 100% alignment agreement for forward and reverse sequencing traces for each sample using the ClustalW sequence alignment program. This indicated that rather than a mixed bacterial population, one major organism was present in each of the samples. *In silico* BLAST analysis of the *16S rRNA* sequence data for sample 2 indicated a 100% homology with the NCTC 11168 *C*. *jejuni* [9]. Sample 3 was identified by BLAST analysis of the sequencing data as *Trichococcus* spp (96% homology), [EU418263]**.** We did not attempt to culture the *Trichococcus* spp, as there was only sufficient sample to do a gDNA extraction for *16S rRNA* PCR but not for culture work. Molecularly the *Trichococcus* spp. was not further characterized due to the lack of specific MLST PCR and sequencing primers for this organism.

### MLST of the *Campylobacter jejuni* isolate

**Figure 2:**
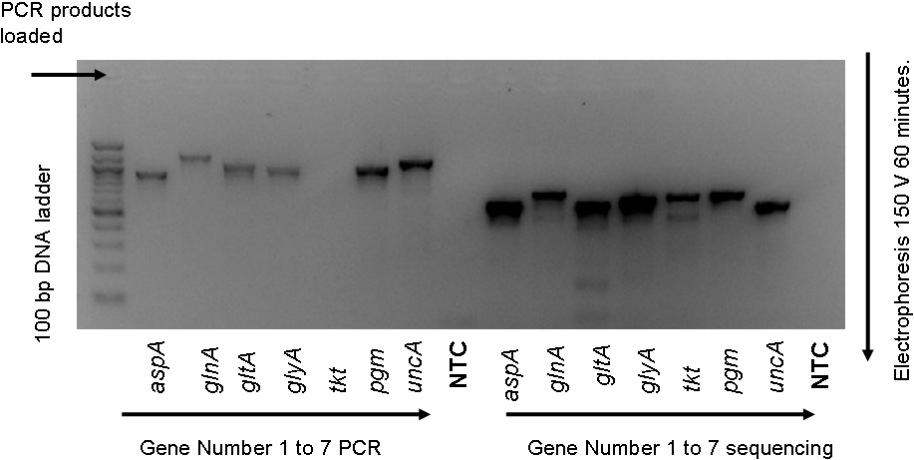
2 % agarose gel, PCR of gDNA extracted from a *C. jejuni* isolate using both PCR and sequencing oligonucleotide primers for *C.jejuni* MLST genes 1 to 7. 2 µl extracted gDNA used in 25 µl PCR reaction with 35×PCR cycles. 3 µl loaded on gel. Image is reverse colour under UV tranillumination (Bio-Rad). NTC = Non-template control, 3 µl of each individual reaction combined and 21 µl loaded on gel.

Six of the seven *C.jejuni* specific MLST genes were successfully amplified by PCR; the fifth *tkt* (transketolase) failed initial PCR amplification but was successfully PCR amplified and sequenced using the sequencing primers in a nested approach (**Figure 2**). All 7 MLST house keeping genes were successfully sequenced in both directions in duplicate, using a direct and nested sequencing approach (Genbank accession numbers EU056829 to EU056835). High quality sequence traces were obtained for all 7 products from both the forward and reverse sequencing reactions and from the nested reactions. ClustalW alignment indicated 100% agreement between forward and reverse reaction sequencing products for all 7 MLST *C.jejuni* specific MLST genes. Sequences resulting from the standard and nested reactions were in 100% agreement.

Submission of the MLST sequence data for all 7 house keeping genes to the campylobacter MLST database, indicated that while all 7 alleles had been described individually previously, they had not been described in the combination that we found. Thus the isolate of *C*. *jejuni* constituted a novel ST not previously described in the literature or the database. The novel ST was designated ST-2928: With an allele profile code; 24, 2, 2, 15, 294, 3, 12, for *aspA*, *glnA*, *uncA*, *glyA*, *pgm*, *tkt*, and *gltA* respectively. The isolate is a member of the previously described *C*.*jejuni* clonal complex ST-443 also isolated and identified in humans [10].

### Bacterial Culture

Culturing an aliquot of the clinical archival sample number 2 under optimum culture conditions for *C*. *jejuni* (in light of the *16S rRNA* data) resulted in florid bacterial growth with morphological characteristics typical of *C.jejuni*. Colonies from the isolate were identified as Gram-negative rods, and biochemically characterized as oxidase, catalase and ninhydrin positive all consistent with *C*. *jejuni*.

## Discussion

We have discovered a novel *C*.*jejuni* isolate in The Gambia (ST-2928) using a molecular based approach as an adjunct to traditional culture and biochemical characterisation protocols. Traditional bacterial culture protocols had failed to isolate the organism in a blood sample when the patient first presented 6.5 years previously; hence the discovery was made as a direct consequence of using molecular protocols. There was no history of treatment with antimicrobials before admission to account for the negative blood culture. Rather, inappropriate incubation of the blood sample at 37°C as opposed to 42°C may explain why the organism was not detected originally in the patient’s blood sample. The laboratory however, had no *a priori* reason to consider culturing this patient’s blood sample at 42°C when the patient first presented. The optimum temperatures for most bacteria that are pathogenic to humans are 35°C–37°C, and hence the temperatures most frequently used to culture organisms in a clinical diagnostic laboratory. Although *Campylobacter* species are not difficult to isolate *per se*, they do require special media, special temperature and atmospheric conditions for growth [11]. *C*.*jejuni* is grown on especially selective agar plates at 42°C, the normal avian body temperature (which constitutes an important source and natural reservoir of the organism). When more appropriate culture conditions were applied, after initial *16S rRNA* sequencing identified the organism in the sample, we obtained florid culture growth, sufficient numbers of bacteria for optimal biochemical testing, and sufficient quantities of DNA for successful MLST analysis of the *C*.*jejuni* isolate.

Our aim was to apply molecular protocols such *16S rRNA* PCR, DNA sequencing and MLST analysis to an archival blood sample from a complex clinical case, which was reported as culture negative for bacterial growth after original presentation. The recent completion of the sequencing of the *C*. *jejuni* genome (NCTC11168) has greatly facilitated advances in molecular based detection and characterization of this pathogen and important public health burden [9]. *C*. *jejuni* is a member of the delta-epsilon group of proteobacteria and is Gram-negative, flagellate, curved or spiral, microaerophilic and motile. *C. jejuni* is the leading cause of bacterial food-borne diarrhoeal disease throughout the world [12] and has been reviewed elsewhere [13]. It has also been described as an important cause of diarrhoeal illness in African children under 5 years of age 14–16. *Campylobacter jejuni* has been divided into two subspecies: *C. jejuni* subsp. *jejuni* and *C. jejuni* subsp. *doylei.* Almost all of the *C. jejuni* strains isolated are *C. jejuni* subsp. *jejuni*. Although subspecies *doylei* strains are isolated only rarely, they differ from subsp. *jejuni* in two important aspects: they are obtained mainly from human clinical samples and are associated more often with bacteraemia [17].

To study the epidemiology of *Campylobacter* infections, multiple typing methods have been developed [18]. The MLST method was developed by Dingle *et al*, to characterize *C*. *jejuni* strains and to identify clonal lineages in this species [19]–[20], [10], [21]–[22]. This method uses genetic variations at multiple chromosomal locations and allows generation of sequence data, which are deposited in internet databases for comparison with DNA sequences of other isolates.

Despite its accuracy, 16S rRNA gene sequence analysis lacks widespread use beyond the large and reference laboratories because of technical and cost considerations, this may be a confounding issue in developing countries such as The Gambia where technological and financial resources are limited. Thus, a future challenge is to incorporate 16S rRNA gene sequencing into routine protocols in concert with traditional microbiological and biochemical testing. The availability of free internet based molecular bioinformatic tools such as search engines and databases for molecular analysis represents a great resource for laboratories within developing countries, using or planning to use molecular protocols in their work.

Amplification and sequencing of the *16S rRNA* has become the method of choice for ‘broad-range’ identification of bacterial species in clinical samples or cultures but for more definitive characterization it is necessary to complete organism specific MLST analysis, and this was the rationale behind our approach in this case. It has been suggested that the clinical interpretation of ‘broad-range’ *16S rRNA* PCR and sequencing results can be problematical *e.g*. the protocol has the potential to detect all bacterial DNA present in a given clinical sample, including non-viable or even degraded organisms. A background level of bacterial DNA might be present in blood of patients without any overt clinical signs of bacteraemia [23]–[24]. The consequences of detection of bacterial DNA in blood in so called ‘healthy’ patients has not been adequately addressed to date. An important *caveat* to these observations is that low levels of bacterial nucleic acids may be due to contamination of reagents and equipment acquired in the process of sample collection and processing or transient bacteraemia. Contamination, however miniscule, has the potential to significantly reduce the sensitivity of the molecular protocols, if not adequately addressed. Contamination is not an issue in the current case as a high load of *C*. *jejuni* was observed without any trace of 16S rDNA in the non-template controls (See **Figure 1**).

While molecular test results are less subjective than those from traditional microbiological/biochemical characterization protocols, it would be premature to advocate replacement of the traditional culture protocols at this juncture with molecular technology. Molecular protocols may however act as an important adjunct to traditional culturing. It may be useful to incorporate the routine use of molecular protocols such as *16S rRNA* PCR and sequencing as an initial screen for microbial pathogens in clinical samples, this would allow more selective and efficient use of traditional culturing for confirmation, the cost of the molecular methods being offset by using less traditional culture associated consumables. Overall, gene amplification and DNA sequencing from culture and clinical material may improve our understanding of microbial pathogenesis and better predict responses to therapy and clinical outcome; they may also provide much needed insight into the complex ecology of mixed infections *in vivo*.

More broadly molecular methods such as these have potentially important clinical and public health applications. From the clinical point of view, key diagnostic possibilities for this patient included serious bacterial infection (including pneumonia, infective diarrhoea, septicaemia), and malaria. In this case the diagnoses were two: there was clear evidence for malaria but the co-existing bacteraemia was missed. While this child recovered from her bacteraemia without specific treatment (though possibly assisted unintentionally by the sufadoxine contained in her malaria therapy), invasive bacterial disease, often unrecognised, is a major killer of children in Africa and globally and improvements in diagnostics are urgently needed to improve outcomes (Mulholland and Adegbola 2005 [25]. This case indicates that molecular methods can improve diagnosis when used in concert with traditional methodologies. A key challenge is to establish more widespread use of molecular protocols in developing world settings. Such advances will be important not only at the bedside but at the population level where better measures of disease burden will contribute significantly to the improvement of public health policy.
